# Resection and Abdominal Wall Reconstruction of a Desmoid Tumor with Endometrioma Features

**DOI:** 10.1155/2016/9453450

**Published:** 2016-05-10

**Authors:** Jaqueline Majors, Nathaniel F. Stoikes, Reza Nejati, Jeremiah L. Deneve

**Affiliations:** ^1^Division of Surgical Oncology, Department of Surgery, University of Tennessee Health Science Center, 910 Madison Avenue, Suite 300, Memphis, TN 38163, USA; ^2^Department of Pathology, University of Tennessee Health Science Center, 910 Madison Avenue, Suite 300, Memphis, TN 38163, USA

## Abstract

Desmoid tumors are rare, musculoaponeurotic mesenchymal origin tumors arising from the proliferation of well-differentiated fibroblasts. Desmoid tumors may arise from any location with the abdominal cavity, abdominal wall and extremity locations being most frequent. We present the case of a 35-year-old female with a history of endometriosis who presented palpable abdominal mass and cyclic abdominal pain. Resection was performed for a presumed desmoid soft tissue tumor. Final pathology demonstrated desmoid histology admixed with abdominal wall endometriosis (endometrioma). This unique pathologic finding has only been rarely reported and is discussed with a brief review of the literature.

## 1. Case Report

**Figure 1:**
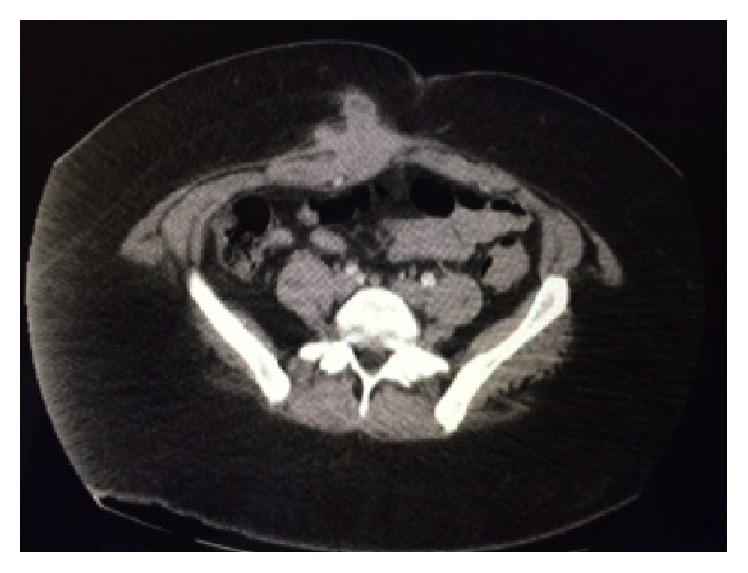
Abdominal wall mass arising from the right rectus abdominis.

**Figure 2:**
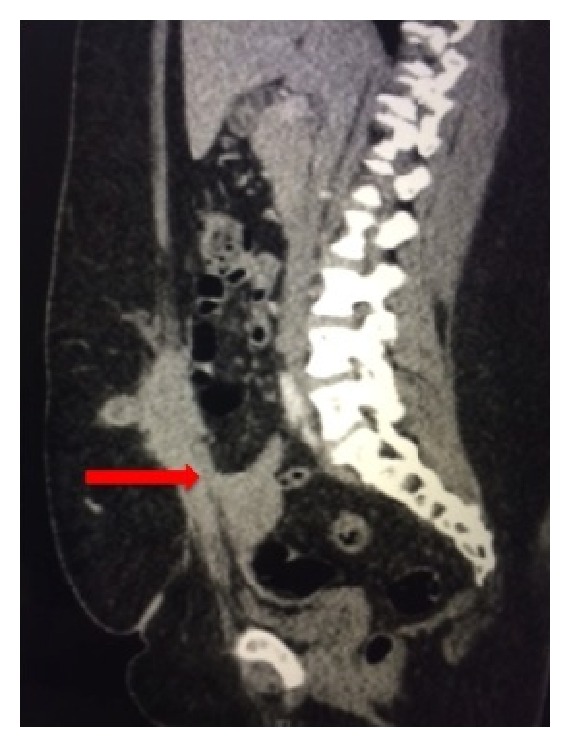
Cross-sectional imaging demonstrating invasion of the abdominal wall mass into the uterus (arrow).

A 35-year-old female presented with sharp, right lower abdominal pain for several months duration. Her pain was cyclical and worse around her menses. She had a previous surgical history of caesarian section 8 years prior and previous gastric bypass for morbid obesity. On physical examination, a moderate sized mass was present above her Pfannenstiel incision on the right abdominal wall that was firm in nature. Cross-sectional imaging demonstrated a right rectus sheath mass measuring 8 cm, which was well circumscribed and suspicious for a desmoid tumor (Figure 1). There was invasion of the fundus of the uterus (Figure 2). Surgery was recommended.

**Figure 3:**
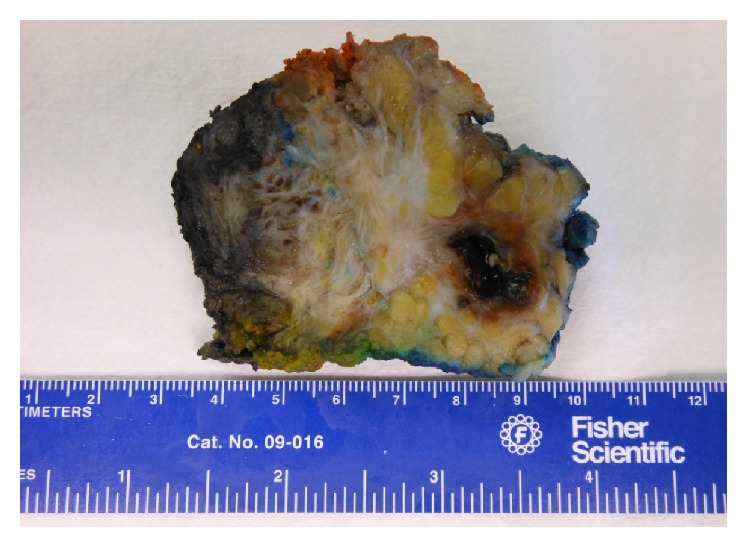
Resected abdominal wall mass with desmoid and endometrioma features.

**Figure 4:**
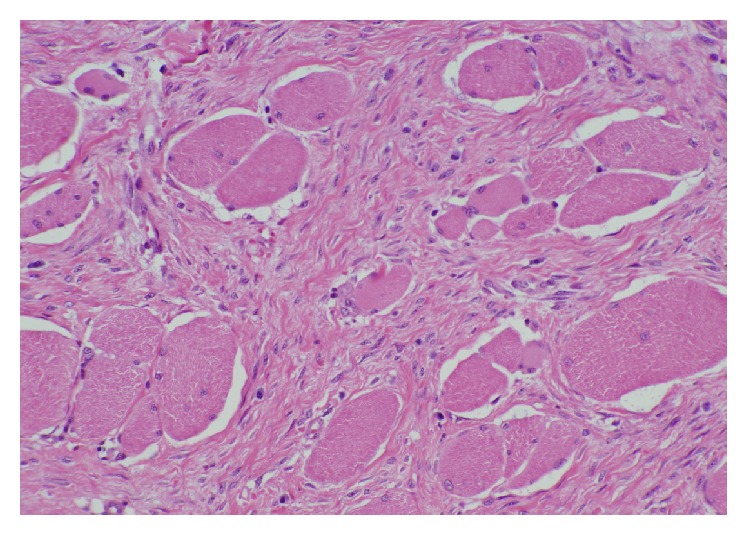
Desmoid tumor with irregular infiltration of bland spindle cells between skeletal muscle cells.

**Figure 5:**
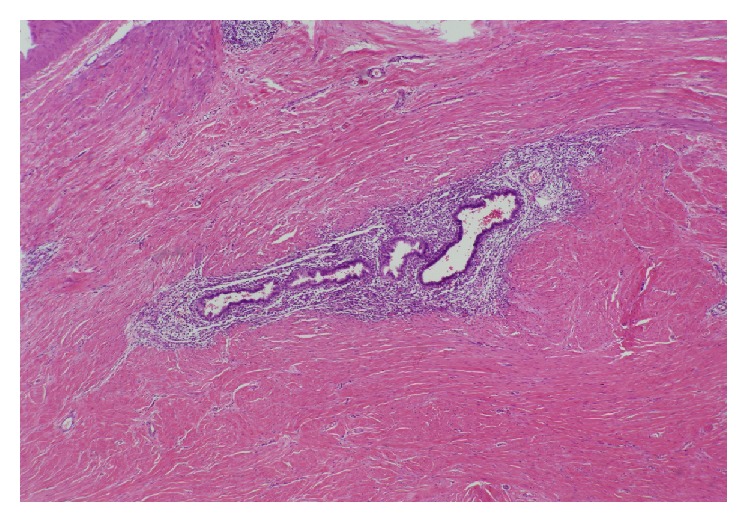
Endometriosis with presence of both endometrial glands and stroma.

At the time of exploration, a paramedian incision was created over the area of the abdominal wall mass. The mass involved the right anterior rectus sheath and rectus abdominis muscle. The posterior rectus sheath was largely spared. Inferiorly, near the previous Pfannenstiel incision, the mass was noted to extend into the peritoneum and was adherent to the fundus of the uterus. The mass was resected off the uterus with grossly negative margins. Intraoperative pathologic frozen section demonstrated abdominal wall endometriosis (Figure 3). The abdominal wall defect was too large to allow primary fascial closure. The 11 × 18 cm abdominal wall defect was repaired in a complex manner. Large myocutaneous flaps were made and a retrorectus absorbable synthetic mesh underlay was combined with a 30 × 30 cm lightweight and macroporous polypropylene mesh onlay repair. In the subcutaneous space, four 19 French Blake drains were placed. The immediate postoperative course was uncomplicated and she was discharged home at day 4. After the drains were removed postoperatively, the patient developed an abdominal wall seroma which was managed with percutaneous aspiration without further sequelae. Final pathology revealed an abdominal wall desmoid admixed with florid endometriosis (Figures 4 and 5). She remains without evidence of recurrence at 6-month follow-up.

## 2. Discussion

Desmoid tumors (aggressive fibromatosis) are rare soft tissue tumors that arise from clonal proliferations of mesenchymal stem cells [1]. These tumors derive their name based on their macroscopic appearance from the Greek origin “desmos,” meaning tendon- or band-like. Histologically they are characterized by a fibromatous, benign proliferation of well-differentiated fibroblast. Desmoid tumors are rare with an estimated incidence of 2–4 cases per million per year and may occur anywhere in the body, with the extremities, abdominal wall, and abdominal cavity being the most common locations [2–4]. A majority of these tumors arise from mutations in the *β*-catenin gene, with a smaller proportion associated with mutations in the APC gene and familial adenomatous polyposis [5, 6].

While these tumors are considered “benign,” the clinical management is highly variable and based on the location and clinical behavior of the primary tumor. In some instances, the lesion remains stable in size and requires no further intervention, while in others the tumors are characterized by rapid growth [7]. Local recurrence rates have been reported as high as 45% which has led some authors to pursue a conservative “wait-and-see” approach to desmoid tumors [8–10]. These tumors generally do not metastasize; however they can exhibit aggressive locoregional behavior and invade into adjacent structures.

Surgical resection with negative pathologic margins is the mainstay of therapy for desmoid tumors. Tumor size, location, patient age, and pathologic margin status have all been associated with higher recurrence. Peng and colleagues reviewed the outcome of 211 patients from a multi-institutional cohort and identified a 53% 5-year recurrence-free survival [2]. Younger patient age, microscopic-positive margin (R1) status, and extra-abdominal tumor location were all associated with increased recurrence. Reports in the literature, however, have not always been congruent with regard to margin status and an association with recurrence. Gronchi et al. reported the outcome of 203 patients (128, primary, and 75, recurrent) with extra-abdominal aggressive fibromatosis at a single institution [11]. Presenting with recurrent disease was the strongest predictor of local failure. Of those presenting with primary desmoid tumors, tumor size and tumor location had prognostic significance while microscopic-positive margin status did not. Postoperative radiation therapy may be considered for those with a positive margin in which relapse or reexcision would be associated with increased morbidity [12]. The role of adjuvant therapy for desmoid tumors has not been standardized. The authors at MD Anderson Cancer Center demonstrated that desmoid tumors are effectively controlled with radiation therapy as an adjunct to surgery when margin-positive resection is anticipated or treating gross disease when surgical resection is not possible [13]. High rates of radiation related complications may develop when treating gross disease; however, this is a prohibitive treatment option for some, especially in situations of truncal or abdominal wall lesions which was the presentation of this patient.

Systemic treatment options exist for locally advanced or unresectable desmoid tumors. Nonsteroidal anti-inflammatory drugs (sulindac or celecoxib), hormonal agents (tamoxifen and toremifene), and chemotherapy have all been described to have an efficacy for desmoid tumors [14–18]. Recently, imatinib and sorafenib have both demonstrated promise against desmoid tumors [19, 20]. Sorafenib use was associated with a partial response in 25% and stable disease in 70% of patients when used as first-line therapy or as salvage therapy for those who progress on cytotoxic chemotherapy [20]. Unfortunately, the optimal strategy to manage patients with desmoid tumors has yet to be defined. Crago and colleagues developed a nomogram to better identify candidates who may be ideally suited for surgical management or those who may benefit from neoadjuvant/systemic therapies and/or nonoperative management [21]. Four hundred ninety-five patients were treated, of which 100 (23%) recurred. The 5-year local recurrence-free survival was 69%. Abdominal wall tumors had the best overall outcome, with a long-term disease-free survival of over 90%. Tumor size, tumors located on the extremities, and patient age were all associated with increased risk of recurrence, while margin status had no impact. The authors concluded that a postoperative nomogram including age, tumor size, and location predicts recurrence and aids in decision-making. They further suggested that systemic therapy might be appropriate for young patients with large extremity tumors while surgery alone may be sufficient for abdominal wall lesions.

Endometriosis is defined as the presence of ectopic endometrial functional glands and stroma. Abdominal wall endometriosis presenting as a mass (endometrioma) has been documented in the scar of prior abdominal incisions or more commonly after caesarian section and is estimated to occur in as many as up to 3.5% of patients with endometriosis after surgical treatment [22, 23]. The likely pathogenesis is related to direct implantation of endometrial tissue during an abdominal surgical procedure. Women with endometriomas can present with a variety of complaints, notably a palpable abdominal mass or cyclic abdominal pain related to menses [24]. Surgical resection is the primary treatment modality for endometriomas. Reconstruction after resection of larger lesions may require mesh reinforcement [25]. Medical therapy with oral contraceptives is largely ineffective [26]. In the situation of the case report described above, we chose to reconstruct the abdominal wall using a complex retrorectus mesh underlay technique with onlay mesh reinforcement with fibrin glue. We have performed this fixation approach routinely at our institution and found it to be less technically demanding with shorter operative times than traditional transfascial repair and less long-term pain [27]. Resection of endometriomas is warranted as there has been an association with other malignancies, such as clear cell adenocarcinoma [28]. Indeed, few isolated reports of abdominal wall endometriosis and desmoid tumor pathology have also been reported [29–31]. Estrogen exposure has also been associated as a risk factor for desmoid tumor formation [32]. The etiology, association, and long-term outcome of desmoid tumor with endometriosis features are currently unknown. At present, the patient presented in this brief report is greater than 6 months without tumor recurrence. Long-term follow-up and multi-institutional reviews are required to better understand the natural history of this rare phenomenon.

## 3. Conclusion

Desmoid tumors are rare, soft tissue tumors arising from mesenchymal origin. Tumors arising from the abdominal wall tend to have a better long-term outcome than those arising from the extremity. Surgery remains the mainstay of therapy with radiation therapy or systemic therapy reserved for those who are unresectable at presentation or in which resection would confer significant function-limiting morbidity. The association of endometriosis features with desmoid tumors is rare. Surgical resection and close follow-up are required for both tumor types. The long-term outcome for this tumor association remains to be determined.
